# Physical activity, sedentary behaviours, and the prevention of endometrial cancer

**DOI:** 10.1038/sj.bjc.6605902

**Published:** 2010-09-28

**Authors:** S C Moore, G L Gierach, A Schatzkin, C E Matthews

**Affiliations:** 1 Division of Cancer Epidemiology and Genetics, National Cancer Institute, 6120 Executive Boulevard, Bethesda, MD 20892, USA

**Keywords:** sedentary behaviour, physical activity, endometrial cancer, epidemiology

## Abstract

Physical activity has been hypothesised to reduce endometrial cancer risk, but this relationship has been difficult to confirm because of a limited number of prospective studies. However, recent publications from five cohort studies, which together comprise 2663 out of 3463 cases in the published literature for analyses of recreational physical activity, may help resolve this question. To synthesise these new data, we conducted a meta-analysis of prospective studies published through to December 2009. We found that physical activity was clearly associated with reduced risk of endometrial cancer, with active women having an approximately 30% lower risk than inactive women. Owing to recent interest in sedentary behaviour, we further investigated sitting time in relation to endometrial cancer risk using data from the NIH-AARP Diet and Health Study. We found that, independent of the level of moderate–vigorous physical activity, greater sitting time was associated with increased endometrial cancer risk. Thus, limiting time in sedentary behaviours may complement increasing level of moderate–vigorous physical activity as a means of reducing endometrial cancer risk. Taken together with the established biological plausibility of this relation, the totality of evidence now convincingly indicates that physical activity prevents or reduces risk of endometrial cancer.

Endometrial cancer is a disease primarily found among affluent developed nations where westernised diets and sedentary lifestyles predominate (Curado *et al*, 2007). Evidence indicates that 40–50% of endometrial cancers may be due to too much body fat, suggesting that energy balance has a critical role in the aetiology of this disease (Calle and Kaaks, 2004). Whether physical activity, a key factor in the regulation of energy balance, also contributes to endometrial cancer risk is less clear. In 2007, three separate publications (Cust *et al*, 2007; World Cancer Research Fund and American Institute for Cancer Research, 2007; Voskuil *et al*, 2007), including the World Cancer Research Fund and American Institute for Cancer Research (WCRF/AICR) report, reviewed the totality of epidemiological evidence on physical activity and endometrial cancer relation, and each concluded that physical activity probably reduces endometrial cancer risk. None of the reviews considered the level of evidence to be ‘convincing’, in large part because of the paucity of prospective data (Moradi *et al*, 1998; Terry *et al*, 1999; Weiderpass *et al*, 2001; Folsom *et al*, 2003; Furberg and Thune, 2003; Schouten *et al*, 2004). However, five large prospective cohort studies have since published findings on the relationship between physical activity and endometrial cancer (Friberg *et al*, 2006; Friedenreich *et al*, 2007; Patel *et al*, 2008; Conroy *et al*, 2009; Gierach *et al*, 2009). Because these data were not included in existing reviews, the reviews' conclusions may no longer reflect the current state of the evidence.

These reviews also did not examine sedentary behaviour (too much sitting) as a risk factor for endometrial cancer. Recent findings indicate that—independent of the amount of moderate-to-vigorous-intensity physical activity undertaken—spending excessive amounts of time sitting is associated with increased risk of obesity and insulin resistance (Healy *et al*, 2008; Helmerhorst *et al*, 2009), premature mortality (Katzmarzyk *et al*, 2009), and possibly endometrial cancer (Friberg *et al*, 2006; Gierach *et al*, 2009). Sedentary behaviours may thus complement moderate–vigorous physical activity. Omission of sedentary behaviours from these reviews could mean that they underestimate the total burden of endometrial cancer due to physical inactivity.

In this review, we reevaluate the biological and epidemiological evidence that low levels of physical activity cause endometrial cancer. We examine the evidence in light of the criteria proposed by the WCRF/AICR report (World Cancer Research Fund and American Institute for Cancer Research, 2007) for a ‘convincing’ causal relationship, which are as follows: (1) experimental evidence that exposure, that is, inadequate physical activity, can biologically cause the cancer, (2) evidence from more than one study type, (3) evidence from at least two prospective studies, (4) no substantial heterogeneity within or between study types, (5) evidence of a dose–response relationship and (6) studies of high quality that allow random or systematic errors to be ruled out as an explanation for study findings. We also examine the evidence that sedentary behaviours are a distinct but complementary potential cause of endometrial cancer.

## Biological evidence linking physical activity with endometrial cancer risk

The endometrium is the lining of the uterus, and it undergoes cyclical regeneration during the menstrual cycle. At the beginning of the menstrual cycle, escalating levels of oestrogen secreted by the ovaries promote production of IGF-1 within uterine stromal cells, resulting in proliferation of the endometrium (Kaaks *et al*, 2002). Later in the cycle, ovulation occurs, and the ovary produces progesterone, which antagonises oestrogen, thereby causing a rapid decline in proliferation. Proliferation of the endometrium is a normal part of the menstrual cycle, but factors that increase oestrogen exposure without compensating progesterone, such as oestrogen-only formulations of menopausal hormone replacement therapy, can increase endometrial cancer risk (Weiderpass *et al*, 1999). Conversely, factors that reduce exposure to bioactive oestrogens may reduce endometrial cancer risk.

Physical activity is hypothesised to decrease endometrial cancer risk because it reduces serum levels of estradiol and increases levels of sex hormone binding globulin (SHBG), the binding protein for estradiol (McTiernan, 2008). These effects of physical activity may be mediated through prevention of weight gain. In postmenopausal women, adipose tissue and the aromatisation of androgen precursors, which occurs within this tissue, is the primary source of oestrogen (Siiteri, 1987). Consequently, women who maintain a healthy body weight tend to have lower circulating oestrogen levels (Calle and Kaaks, 2004).

Chronic hyperinsulinaemia is an additional and important biological factor that influences endometrial cancer risk (Gunter *et al*, 2008). Hyperinsulinaemia may promote endometrial carcinogenesis by stimulating endometrial proliferation directly, or indirectly by increasing IGF-1 levels within the endometrium and decreasing levels of its binding proteins (e.g., IGFBP) (Kaaks *et al*, 2002). Hyperinsulinaemia could also increase levels of bioavailable oestrogens, as it has been linked to decreased levels of the oestrogen-binding protein SHBG (Kaaks *et al*, 2002). Physical activity of a moderate-to-vigorous intensity could reduce risk of endometrial cancer because it improves insulin sensitivity and reduces circulating levels of insulin (McTiernan, 2008). Sitting time may also be linked to endometrial cancer risk through these insulin-related mechanisms, as excessive sitting is associated with low levels of energy expenditure (Owen *et al*, 2009), as well as with weight gain (Blanck *et al*, 2007) and insulin resistance (Healy *et al*, 2008).

## Epidemiological evidence linking physical activity with endometrial cancer risk

The epidemiological evidence linking physical activity with endometrial cancer risk was last reviewed in three separate reports published in 2007 (Cust *et al*, 2007; Voskuil *et al*, 2007; World Cancer Research Fund and American Institute for Cancer Research, 2007). Each review reached the same conclusion, that is, that physical activity probably reduces endometrial cancer risk, with active women at a 20–40% reduced risk of endometrial cancer relative to inactive women. An important limitation of these reviews is that the evidence evaluated was based primarily on case–control studies, which can be susceptible to recall bias. If cases underestimated their physical activity level relative to controls, physical activity could appear to protect against endometrial cancer even in the absence of a true association.

In the WCRF/AICR report, a total of four prospective studies of recreational physical activity (Terry *et al*, 1999; Folsom *et al*, 2003; Furberg and Thune, 2003; Schouten *et al*, 2004), comprising 800 cases, and three prospective studies of occupational physical activity (Weiderpass *et al*, 1999, 2001; Furberg and Thune, 2003), comprising 8250 endometrial cancer cases, were available for review. The occupational physical activity studies were well powered and each reported that physical activity was inversely associated with endometrial cancer risk. In contrast, the recreational physical activity studies had low statistical power, even in aggregate, and the findings were suggestive but did not allow chance to be ruled out as an explanation for study findings.

The WCRF/AICR report described the evidence that physical activity protects against endometrial cancer risk as ‘probable’ rather than ‘convincing’, and they specifically note that the evidence was mostly based on case–control studies (World Cancer Research Fund and American Institute for Cancer Research, 2007). Since publication of the WCRF/AICR report in 2007, five large prospective cohort studies have examined physical activity in relation to endometrial cancer risk (Friberg *et al*, 2006; Friedenreich *et al*, 2007; Patel *et al*, 2008; Conroy *et al*, 2009; Gierach *et al*, 2009). For recreational physical activity, these studies added 2663 cases (Friberg *et al*, 2006; Friedenreich *et al*, 2007; Patel *et al*, 2008; Conroy *et al*, 2009; Gierach *et al*, 2009), increasing the total from 800 to 3463 cases.

## Meta-analysis incorporating recent findings

To determine whether these new findings would allow for firmer conclusions regarding the relationship between physical activity and endometrial cancer, we updated the WCRF/AICR meta-analysis of prospective cohort studies. PubMed was searched for prospective studies published in English through to December 2009 that examined volume of physical activity using the following search terms: (‘physical activity’ or ‘exercise’ or ‘physical fitness’ or ‘sedentary’) and (‘endometrial cancer’ or ‘uterine cancer’). References from relevant publications were hand searched for additional articles.

We identified nine prospective cohort studies of recreational physical activity and five of occupational physical activity. The multivariate relative risk (RR) and 95% confidence intervals of endometrial cancer risk for the highest *vs* lowest level of physical activity were abstracted. Most studies of recreational physical activity investigated time spent in varying leisure time exercise activities, for example, walking, cycling and/or sports (Folsom *et al*, 2003; Schouten *et al*, 2004; Friberg *et al*, 2006; Friedenreich *et al*, 2007; Patel *et al*, 2008; Conroy *et al*, 2009), but a few focused on intense exercise activities such as running, or which cause a sweat (Terry *et al*, 1999; Furberg and Thune, 2003; Gierach *et al*, 2009). The reference category of physical activity in these studies included those with little or no recreational physical activity, except for one study that explicitly excluded those with no recreational physical activity from the reference category (Patel *et al*, 2008). Studies of occupational physical activity imputed physical activity level on the basis of job codes (Moradi *et al*, 1998; Weiderpass *et al*, 2001), or by asking study participants to classify the intensity of their occupation (Furberg and Thune, 2003; Friberg *et al*, 2006; Friedenreich *et al*, 2007); the reference category for these studies was a sedentary occupation, for example, deskjob.

Where possible, we used results from models without adjusting for BMI because at least a part of physical activity's effect on endometrial cancer risk is anticipated to be through reductions in adiposity; thus, BMI should not be adjusted for in statistical models. Results of models with and without adjustment for BMI were available only in Friberg *et al* (2006), Patel *et al* (2008), and Gierach *et al* (2009), each published after the AICR review. All other studies presented only results adjusted for BMI, except the study by Folsom *et al*, which presented age-adjusted results only.

We calculated pooled RR estimates and 95% confidence intervals using a random-effects model that weighed individual study-specific RRs by the inverse of the sum of their variance. We assessed study-to-study variability in RRs using tau-squared (*τ*^2^) (DerSimonian and Laird, 1986), and by inspection of relative risks. All analyses were conducted using Stata 9.0.

**Figure 1:**
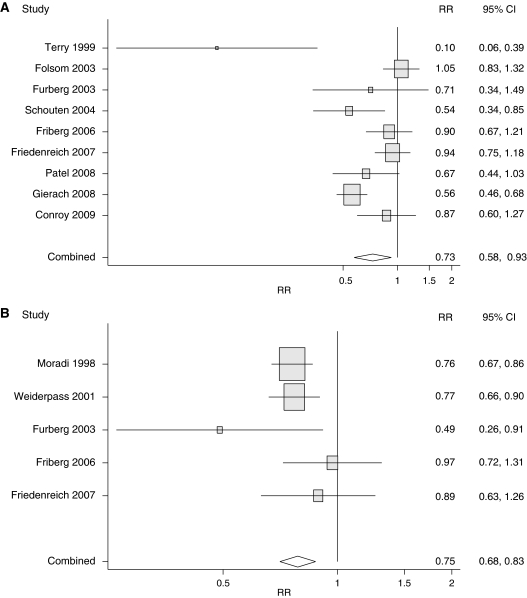
(**A**) Relative risk (RR) and 95% confidence intervals (CI) of endometrial cancer according to the highest *vs* the lowest level of recreational physical activity. Relative risks were obtained from multivariate adjusted models, except Folsom *et al* (2003), which presented only age-adjusted results. For Friberg *et al* (12), Patel *et al* (14), and Gierach *et al* (15), we used results from models without adjustment for BMI, although BMI-adjusted results were available in separate models in these publications. In a sensitivity analysis, we examined results when using only RRs adjusted for BMI (including all studies, except Folsom *et al*). In these models, there was modest attenuation of relative risks, but an inverse association was still evident (pooled RR=0.78; 95% CI=0.63, 0.95). (**B**) Relative risk (RR) and 95% confidence intervals (CI) of endometrial cancer according to the highest *vs* lowest level of occupational physical activity.

Comparing women with the most recreational physical activity with those with the least, the pooled RR and 95% confidence intervals (CI) for endometrial cancer were 0.73 (0.58, 0.93) (Figure 1). The confidence intervals for the pooled RR do not contain 1.0, allowing us to conclude that chance is an unlikely explanation for study findings. In the WCRF/AICR report, the pooled RR and 95% CI were 0.57 (0.30, 1.09); the wide confidence interval in this previous review did not allow chance to be ruled out.

As a sensitivity analysis, we examined results that were adjusted for BMI (all studies except by Folsom *et al*). These results were somewhat attenuated, but an inverse association was still plainly evident (pooled RR=0.78; 95% CI=0.63, 0.95). We also examined the influence of Gierach *et al* (the largest study) and Terry *et al* (the study with the strongest inverse relation) by omitting each and rerunning the analysis. The results remained statistically significant even after omitting Gierach *et al* (pooled RR=0.78; 95% CI=0.62, 0.98) and Terry *et al* (pooled RR=0.78; 95% CI=0.63, 0.96).

There was no substantial heterogeneity of study findings (*τ*^2^=0.08). All but one of the studies reported an RR that was below 1.0. The outlying study, by Folsom *et al*, was adjusted only for age, and it is unclear whether findings were confounded by important endometrial cancer-risk factors such as parity and/or smoking history. Of the seven studies in our review that included a statistical test for trend (Terry *et al*, 1999; Furberg and Thune, 2003; Schouten *et al*, 2004; Friedenreich *et al*, 2007; Patel *et al*, 2008; Conroy *et al*, 2009; Gierach *et al*, 2009), four found a significant dose–response relationship (Terry *et al*, 1999; Schouten *et al*, 2004; Patel *et al*, 2008; Gierach *et al*, 2009).

We assessed whether potential confounders were appropriately controlled for in these studies. All but the two smallest studies (Terry *et al*, 1999; Furberg and Thune, 2003) properly excluded women with hysterectomies from analysis. Aside from Folsom *et al* (discussed above), all remaining studies adjusted for parity, and only two of the eight studies failed to adjust for smoking status (Terry *et al*, 1999; Folsom *et al*, 2003). Adjustment for age at menarche and age at menopause was carried out in three of the eight studies (Schouten *et al*, 2004; Friedenreich *et al*, 2007; Patel *et al*, 2008) and adjustment for hormone replacement therapy was carried out in four studies (Friedenreich *et al*, 2007; Patel *et al*, 2008; Gierach *et al*, 2009). However, relative risks did not seem to differ on the basis of whether studies adjusted for these factors. On balance, our findings do not seem to be explained by known potential confounders.

Unlike the results for recreational physical activity, results for occupational physical activity remain largely unchanged when incorporating the findings of recently published studies. Comparing women with the most occupational activity with those with the least occupational activity, we found a pooled RR and 95% CIs of 0.79 (0.71, 0.88). The WCRF/AICR report found a similar pooled RR and 95% CIs of 0.75 (0.68, 0.83).

Considering together the biological and epidemiological data, inadequate physical activity now seem to meet the criteria for a convincing causal relationship with endometrial cancer. Thus, corresponding to the AICR/WCRF criteria presented earlier (1) it is well established that physical activity helps to prevent weight gain, improves insulin sensitivity and may reduce circulating oestrogen levels; these biological factors are relevant to endometrial cancer risk. (2) An accumulation of case control studies and (3) multiple prospective studies show that increased recreational and occupational physical activity levels are associated with reduced endometrial cancer risk. (4) Data for both occupational and recreational physical activity consistently demonstrate an inverse association, with all but one study reporting a relative risk below 1.0. (5) Several studies report a linear dose–response relationship. Finally, (6) the prospective design of recent studies limits the potential for selection bias and these studies have been carefully controlled for important demographic and lifestyle factors, such as age and use of hormonal therapies.

On the basis of our own data from the NIH-AARP Diet and Health Study data, we preliminarily estimate, using the Walter formula for population attributable risk (Szklo and Nieto, 2004), that 22% of endometrial cancers could have been prevented if all NIH-AARP Diet and Health Study participants had exercised vigorously (defined as any bout of 20 or more minutes of activity at work or at home that caused increased breathing, heart rate or a sweat) at least five times per week. This estimate may represent the upper bound for population attributable risk, as the magnitude of physical activity and endometrial cancer relation in this study was greater than that of most others.

## Future directions: sedentary behaviours and endometrial cancer

For much of the past 50 years, the study of physical activity epidemiology has focused on moderate-to-vigorous-intensity physical activity (MVPA) and its relationship with morbidity and premature mortality. However, there has been recent and increasing interest in sedentary behaviours as a distinct exposure.

Sedentary behaviours are pursuits carried out while sitting or reclining and that do not increase energy expenditure substantially above the resting level (e.g., 1.0–1.5 times the resting level). Independent of moderate–vigorous exercise undertaken, excessive time spent sitting has been associated with reduced levels of light-intensity activity and less energy expenditure (Owen *et al*, 2009), as well as with poor metabolic health (Healy *et al*, 2008), and possibly increased risk for certain cancers. These associations have been demonstrated not only for self-reported sitting time but also for objectively measured sedentary time (Healy *et al*, 2008). Even among adults who meet the recommended 30 min or more of moderate–vigorous physical activity per day (e.g., by walking, playing sports, or exercising in a fitness centre), there seems to be deleterious metabolic consequences to sitting most of the rest of the day (Owen *et al*, 2010). These consequences may stem from the tendency for sitting to displace light-intensity activities, such as time spent standing or in intermittent ambulation (Owen *et al*, 2010). Displacement of 1 h of light-intensity activity by 1 h of sedentary activity results in an estimated loss of 1 MET-hday^−1^ (or 1 kcalkg^−1^day^−1^) of physical activity energy expenditure, equivalent to about 15 min per day of walking (Owen *et al*, 2010). Alternately, sitting time may have its own distinct insidious physiology. Prolonged periods of sitting result in reduced activity of skeletal muscle lipoprotein lipase (LPL)—an enzyme essential for triglyceride catabolism and HDL regulation—and inhibition of glucose uptake (Owen *et al*, 2010).

To date, three prospective studies have examined sitting time in relation to endometrial cancer risk. Friberg *et al* found that women who sat for 5 or more hours per day had increased risk of endometrial cancer relative to women who sat less than that; the RR (95% CI) for endometrial cancer was 1.80 (1.14–2.83) (Friberg *et al*, 2006). Patel *et al* compared women who sat 6+ h *vs* those who sat for less than 3 h per day and reported increased endometrial cancer risk; RR=1.40 (95% CI=1.03–1.89) (Patel *et al*, 2008). Gierach *et al* examined women who sat for 7+ h *vs* those who sat for less than 3 h per day and reported increased risk, with an RR of 1.56 and a 95% confidence interval of 1.22–1.99 (Gierach *et al*, 2009). Whether sedentary behaviours contribute to risk independently from MVPA is not clear from these studies, as two of them (Patel *et al*, 2008; Gierach *et al*, 2009) did not adjust for level of MVPA. Indirect support for a link between sitting time and endometrial cancer risk also comes from studies of occupational physical activity, as they examine risk conferred by sedentary *vs* non-sedentary occupations. The increased risk among women with sedentary jobs is very clear in these studies.

To further investigate the role of sedentary behaviours in endometrial cancer aetiology, we updated the analysis by Gierach *et al* from the NIH-AARP Diet and Health Study by extending cancer follow-up through 31 December 2006 (Gierach *et al*, 2009). Briefly, the study consisted of AARP members aged 50–71 years residing in 1 of 8 US states (CA, FL, PA, NJ, NC, LA, GA and MI). Participation in vigorous-intensity activities during the previous year was assessed on the basis of a baseline questionnaire and time spent sitting per day during the previous year was assessed on the basis of a second questionnaire. Our analysis included the 69 648 women studied by Gierach *et al* who reported both their level of exercise and their time spent sitting per day, of whom 888 were diagnosed with endometrial cancer. All women included in the analysis had an intact uterus, no personal history of cancer, and no missing or extreme values of BMI.

In Table 1, we show that endometrial cancer risk is positively associated with time spent sitting (*P* for trend <0.01), as previously reported by Gierach *et al.* Adjustment for vigorous-intensity physical activity modestly attenuates RRs but sitting time still has a dose–response relationship with endometrial cancer risk (*P* for trend <0.01). Among women who were active, that is, women who engaged in vigorous exercise three or more times per week, as well as among women who exercised less frequently, sitting time was associated with increased endometrial cancer risk (*P* for trend <0.01). In a joint effects analysis, women who were inactive (as defined above) and who sat for 9+ h per day had twice the risk of endometrial cancer of active women who sat fewer than 3 h per day (RR=2.14; 95% CI=1.48, 3.10).

As a sensitivity analysis, we reran our analysis for all women with adjustment for BMI, as well as physical activity level (Table 1). Adjustment for BMI attenuated results substantially. Women who sat for 9+ h *vs* those who sat for fewer than 3 h per day had an RR for endometrial cancer of 1.15 (95% CI=0.87–1.53) as compared with the previous RR of 1.45 (95% CI=1.10–1.92), although the trend remained statistically significant (*P* for trend <0.01). Because sitting time is hypothesised to affect endometrial cancer risk at least partly through its effects on body weight, these results may be conservative.

Our data, and that of others (Friberg *et al*, 2006; Patel *et al*, 2008), suggest that sitting time contributes to endometrial cancer risk, independent from one's participation in MVPA. We earlier estimated that as many as 22% of endometrial cancers could be avoided if women exercised vigorously five or more times per week. However, if all women had both exercised at this level *and* sat for 4 or fewer hours per day, then 34% of endometrial cancers could have been avoided. Thus, the incremental contribution of sedentary behaviours to the population level burden of endometrial cancer risk may be substantial.

## Conclusion

As recently as 2007, prospective data on the relationship between recreational physical activity and endometrial cancer were quite limited. Since then, new prospective studies have helped to redefine knowledge on this epidemiological relation, and aggregated results now clearly indicate a link between higher levels of physical activity and reduced endometrial cancer risk. Taken together with previous knowledge of the biological mechanisms that can explain this relation, these findings indicate that there is sufficient evidence to rule that physical activity has a ‘convincing’ causal relationship with endometrial cancer risk.

Furthermore, an additional physical activity-related exposure, that of excessive sitting time, is emerging as a potential endometrial cancer risk factor. On the basis of initial evidence, excessive sitting time seems to contribute to endometrial cancer risk independently of MVPA. Future studies are needed to confirm the hypothesis that sitting time is independently associated with endometrial cancer and to refine estimates of the proportion of endometrial cancers that could be prevented by increasing participation in moderate–vigorous physical activity and reducing sitting time.

## Figures and Tables

**Table 1:** Multivariate relative risks (RR) and 95% confidence intervals (CI) of endometrial cancer in relation to time spent sitting per day and joint categories of sitting and vigorous intensity physical activity among 69 648 women in the NIH-AARP Diet and Health Study

	**Time spent sitting per day**					
	**<3 h**	**3–4 h**	**5–6 h**	**7–8 h**	**9+ h**	***P*_trend_**
*All women*						
No. of cases	150	232	274	152	80	
Multivariate^a^ RR (95% CI)	1.0 (ref)	1.15 (0.94, 1.42)	1.48 (1.22, 1.81)	1.61 (1.28, 2.02)	1.59 (1.21, 2.10)	<0.01
Multivariate^b^ RR (95% CI)	1.0 (ref)	1.13 (0.92, 1.39)	1.43 (1.17, 1.75)	1.52 (1.21, 1.91)	1.45 (1.10, 1.92)	<0.01
Multivariate^c^ RR (95% CI)	1.0 (ref)	1.07 (0.87, 1.32)	1.29 (1.05, 1.57)	1.33 (1.05, 1.67)	1.15 (0.87, 1.53)	<0.01
						
*Active*^d^ *women*						
No. of cases	74	94	97	51	16	
Multivariate^b^ RR (95% CI)	1.0 (ref)	1.04 (0.77, 1.42)	1.34 (0.99, 1.81)	1.54 (1.07, 2.21)	1.19 (0.69, 2.05)	<0.01
Multivariate^c^ RR (95% CI)	1.0 (ref)	0.99 (0.73, 1.35)	1.22 (0.90, 1.66)	1.37 (0.96, 1.97)	0.98 (0.57, 1.70)	0.08
						
*Inactive women*						
No. of cases	76	138	177	101	64	
Multivariate^b^ RR (95% CI)	1.0 (ref)	1.21 (0.92, 1.61)	1.51 (1.16, 1.98)	1.55 (1.15, 2.10)	1.60 (1.14, 2.24)	<0.01
Multivariate^c^ RR (95% CI)	1.0 (ref)	1.14 (0.86, 1.51)	1.35 (1.03, 1.76)	1.33 (0.99, 1.80)	1.25 (0.89, 1.76)	0.04
						
*Joint effects of vigorous physical activity and sitting time*						
Active women	1.0 (ref)	1.04 (0.77, 1.42)	1.34 (0.99, 1.81)	1.55 (1.08, 2.21)	1.19 (0.69, 2.05)	
Inactive women	1.35 (0.94, 1.93)	1.63 (1.18, 2.26)	2.03 (1.48, 2.79)	2.09 (1.49, 2.93)	2.14 (1.48, 3.10)	

a Multivariate models are adjusted for age (continuous), race (white *vs* other/unknown), smoking status (never, former, current or unknown), parity (nulliparous, one, two, ⩾three births or unknown), ever use of contraceptives (no, yes, unknown), age at menopause (premenopausal, natural menopause at <45, 45–49, 50–54, or ⩾55 years of age or unknown age at menopause), and hormone therapy formulation (never used, estrogen therapy use, estrogen plus progestin therapy use or unknown hormone therapy use).

b Relative risks are additionally adjusted for level of vigorous physical activity (never/rarely, 1–3 times/month, 1–2 times/week, 3–4 times/week, 5+ times/week).

c Relative risks are further adjusted for body mass index.

d Active women are those who engaged in vigorous physical activity for a bout of 20 or more minutes at least three times per week. Inactive women did vigorous physical activity fewer than three times per week.
